# Genetic Evaluation of Pepper Mild Mottle Virus as an Indicator in Water Quality Monitoring and Human Fecal Contamination in Swedish Waters

**DOI:** 10.1007/s12560-026-09690-6

**Published:** 2026-04-17

**Authors:** Marianela Patzi Churqui, Fredy Saguti, Timur Tunovic, Ambjörn Kärmander, Martin Lagging, Kristina Nyström, Hao Wang

**Affiliations:** 1 Department of Infectious Diseases, Institute of Biomedicine, University of Gothenburg, Gothenburg, Sweden; 2 Department of Clinical Microbiology, Sahlgrenska University Hospital, Region Västra Götaland, Gothenburg, Sweden

**Keywords:** Viral indicator, Wastewater, Surface water, Drinking water treatment, Environmental surveillance

## Abstract

Pepper mild mottle virus (PMMoV) has emerged as a promising viral indicator for human fecal contamination and evaluating viral removal efficiency in water treatment systems. However, few studies have examined this potential from a genetic perspective over extended timescales. This study investigated PMMoV presence and genetic diversity across multiple water environments in Sweden, including wastewater, lake water, and raw water used for drinking water production, collected between 2016 and 2024. PMMoV was consistently detected in both wastewater and lake water, showing relatively stable concentrations in wastewater between years, and with levels typically 4–7 log₁₀ higher than in lake water. Comparative genotyping of the coat protein (CP) and replication-associated (RP) regions for wastewater samples showed no significant differences. We successfully sequenced 67 isolates from wastewater, 16 from lake water, and 2 from source water at the CP region. Sequence analysis revealed extremely high nucleotide identities (> 96%) with minimal divergence between samples collected six years apart or from different water types. Phylogenetic comparisons with global reference sequences confirmed high genetic conservation worldwide. These findings demonstrate the remarkable genetic stability of PMMoV over time and geography, supporting its reliability as a viral process indicator for water treatment systems. However, the potential for PMMoV to originate from non-human sources suggests it should not be used as a standalone marker for human-specific fecal contamination. Overall, this study highlights the global genetic conservation of PMMoV and its utility for long-term environmental surveillance and water quality assessment.

**Supplementary Information:**

The online version contains supplementary material available at 10.1007/s12560-026-09690-6.

## Introduction

Water safety is critical to public health, as waterborne pathogens, particularly viruses, pose significant global health risks. Traditional bacterial indicators are widely used to assess water quality but often fail to correlate reliably with the presence of viral pathogens. This gap highlights the urgent need for robust viral surrogates to monitor human fecal contamination and evaluate viral removal efficiency in drinking water treatment plants (DWTPs). Conventionally, coliphages (somatic and F-specific), which infect *Escherichia coli*, have been applied as surrogates and potential indicators of fecal contamination (McMinn et al., 2017). However, their utility is limited by challenges such as possible replication in environmental waters and labor-intensive detection and enumeration methods (Jofre et al., 2016). These limitations have driven interest in alternative viral indicators, such as pepper mild mottle virus (PMMoV) and crAssphage, which show promise as more reliable markers of human fecal contamination and as tools to better assess DWTP performance (Malla et al., 2019; Tandukar et al., 2020).

Among the emerging viral indicators, PMMoV has attracted particular attention. PMMoV is a plant virus that naturally infects pepper plants (*Capsicum spp.*) (Ochar et al., 2023), but it is also widely detected in human feces due to the consumption of pepper-containing foods. Several characteristics make PMMoV a valuable candidate for a viral indicator of human fecal contamination and as a tool for evaluating water treatment performance. Firstly, this virus is abundant across diverse water bodies, from wastewater to surface water, even in tap water (Canh et al., 2021; Djoulissa et al., 2025). This ubiquity, combined with its global presence, enables a broad applicability. Second, it is resistant to environmental conditions and water treatment processes, making it a robust surrogate for human enteric viruses (Bacnik et al., 2020; Kitajima et al., 2018). Finally, the ability to rapidly detect PMMoV using molecular methods, such as qPCR, further strengthens its utility as a practical and efficient indicator in water quality monitoring.

Despite these promising attributes, several knowledge gaps persist in the global application of PMMoV as a viral indicator. Although its widespread occurrence is well established, as an RNA virus with moderate evolutionary rates (Guan et al., 2018; Holmes, 2003), its genetic variability across environmental contexts and geographic regions, particularly in northern climates and low population density settings, are rarely investigated. Moreover, while PMMoV is primarily associated with human fecal sources, contributions from non-fecal and non-human origins, such as infected plants via agricultural runoff, certain animal species like geese, chicken, cows, and seagulls (Gyawali et al., 2019; Hamza et al., 2011), may introduce background levels that complicate human fecal specific monitoring. In addition, studies have reported inconsistent findings regarding its viral removal during water treatment. In some cases, PMMoV showed a removal level comparable to human enteric viruses (Shirasaki et al., 2017, 2018), while in others it had a lower removal level due to greater persistence than human viruses (Rachmadi et al., 2016; Shirakawa et al., 2022), raising concerns about its representativeness as a surrogate for human health risks. Addressing these uncertainties is essential to ensure the reliable integration of PMMoV into water quality monitoring frameworks at both regional and global scales.

In this study, we address some of these critical gaps by investigating the presence, genetic conservation, and environmental dissemination of PMMoV across diverse water types in Sweden, including influent wastewater, lake waters, and raw water used for drinking water production. Sweden provides a relevant setting due to its cold temperate climate, which may prolong viral persistence in environmental waters. Using a longitudinal dataset spanning eight years enables the differentiation of short-term genetic fluctuations from long-term evolutionary trends. Furthermore, by comparing PMMoV sequences from high-load wastewater to low-titer lake and drinking water sources waters, we assess viral genetic conservation during transmission. Overall, this study evaluates the suitability of PMMoV as a robust indicator of human fecal contamination and water treatment performance.

## Materials and Methods

### Sampling of Wastewater, Lake Water, and Source Water for Drinking Water Production

Archived flow-weighed influent wastewater was collected at two time periods: October 3, 2016-January 15, 2018 (period 1), and August 7, 2023-April 1, 2024 (period 2), from the Rya wastewater treatment plant (WWTP) in Gothenburg, Sweden. In period 1, weekly (samples from 2016) and bi-weekly (samples from 2017 and early 2018) wastewater samples were originally collected to evaluate viral removal efficiency in the Rya WWTP. In period 2, weekly wastewater samples were collected to monitor the presence of SARS-CoV-2 and several human enteric viruses. At those time points, the presence and genetic diversity of PMMoV were not investigated. Sampling location, treatment processes, sampling volume and protocols for period 1 have been previously described (Churqui et al., 2024; Wang et al., 2020). Sampling information for period 2, including collection dates and volumes, has not been previously published and is provided in Supplementary Table 1. In total, 74 influent wastewater samples were included in this analysis, including 40 from period 1 and 34 from period 2.

Approximately one-liter grab samples were collected monthly from four lakes located near residential areas in Gothenburg between November 15, 2023, and December 20, 2024. Lake Delsjön is also the source water for drinking water in Gothenburg, while the other lakes (Slottsskogen, Axlemossen, and Högsbo) are close to human recreational activities. There is no regular swimming in Slottsskogen, Axlemossen and Högsbo, only in Delsjön where there is a public beach. Sampling was not possible during cold months when lakes were frozen. In total, 55 lake samples were collected and analyzed. Detailed information on collection sites, dates, and volumes is provided in Supplementary Table 2.

Raw water used for drinking water production was collected from six Swedish DWTPs located in southern and central Sweden. Each DWTP was sampled twice between March 2021 and February 2022, with at least 6 months apart. The sampled DWTPs were Lovö vattenverk and Görvälnverket that take source water from Lake Mälaren, Vombverket take water from Lake Vomb, Borgs vattenverk takes water from Motala Stream, Kvarnagårdens vattenverk takes water from Lake Neden (80%) and groundwater (20%), Vombverket takes water from Lake Vomb, and Ringsjöverket takes water from Lake Bolmen. In total, 12 raw water samples were collected. Sample volumes ranged from 20 to 7,085 L, depending on the DWTP and sampling conditions. Detailed information on sampling locations, dates, and volumes is provided in Supplementary Table 3.

### Concentration of Viruses from Sampled Water

Viruses from influent wastewater collected during period 1 were concentrated by the skimmed-milk flocculation method, as previously described (Wang et al., 2020). For period 2, an in-house method using NanoCeram filter followed by ultracentrifugation was applied (Wang et al., 2018). Viruses from lake samples were concentrated using Amicon ultrafiltration method. Briefly, approximately 1 L of each lake water sample was either centrifuged at 6,000 rpm or filtered through 10 µM nylon net filters to remove larger debris and then filtered using a Sartobran 300 microfilter (0.65 and 0.45 µM; Sartorius, Goettingen, Germany). To further concentrate the viruses, ultrafiltration was performed with a 100 kDa filter attached to an Amicon stir cell (Merck, Darmstadt, Germany). The total volume of water collected after ultrafiltration was 10 mL. Following the original analyses, all remaining concentrated materials were stored at -80 °C until reanalyzed in the present study.

For raw water from drinking water treatment plants, electropositive charged NanoCeram filters were connected directly to incoming raw water pipes at Vombverket, Borgs vattenverk, Ringsjöverket, and Kvarnagårdens vattenverk. After collection, the filters were shipped to the Clinical Microbiological Laboratory (CML) at Sahlgrenska University Hospital, Gothenburg, for secondary concentration. For Görvälnverket and Lovö vattenverk, 20 L of raw water was transported to the CML, where viruses were concentrated using NanoCeram filtration. In all cases, after primary concentration with NanoCeram, samples were further concentrated by ultracentrifugation, as described previously (Wang et al., 2018).

### Detection of PMMoV by qPCR and Nested-PCR

Viral nucleic acids were extracted from concentrated samples using either the DNeasy blood and tissue kit (Qiagen, Hilden, Germany) for wastewater and lake water, or the PureLink RNA Mini kit (ThermoFisher Scientific, Carlsbad, CA, USA) for raw drinking water samples, following the manufacturer’s instructions.

PMMoV was detected and quantified by a one-step RT-qPCR assay. Each 20 µL reaction contained 5 µL of extracted nucleic acids, 4× UltraPlex 1-Step ToughMix (Quantabio, Beverly, USA), 0.375 µM of each primer, 0.25 µM probe, and 9 µL nuclease-free water (Sigma-Aldrich, St. Louis, USA). Primer and probe sequences are listed in Table 1. The qPCR reaction was performed on a QuantStudio 5 Real-Time PCR System (Applied Biosystems, Foster City, CA, USA) with an initial reverse transcription cycle of 50 °C for 10 min and 95 °C for 10 min, followed by 45 cycles of 95 °C for 10 s and 60 °C for 1 min. All samples were run in duplicate. In each run, nuclease-free water was used as a negative control. A synthetic plasmid containing the PMMoV target region (Eurofins Genomics, Ebersberg, Germany) was used as a positive control. Five 10-fold serial dilutions of the plasmid were used to generate standard curves, from which PMMoV concentrations were calculated and expressed as genome copies per milliliter (copies/mL) based on the average Ct values.

For the amplification of PMMoV genome, extracted RNA was reverse-transcribed into cDNA using the High-Capacity cDNA Reverse Transcription Kit (Applied Biosystems). The 20 µL reaction mix contained 10 µL of template RNA, 1× RT buffer, 1× random primers, 4 mM dNTPs, 1 µL RNase inhibitor, 1 µL MultiScribe reverse transcriptase, and 3.2 µL nuclease-free water. The amplification program consisted of 25 °C for 10 min, 37 °C for 120 min, and 85 °C for 5 min. Two nested PCR assays were then developed to target different genomic regions of PMMoV. The first PCR targeted the coat protein (cp) region, adapting primers from several published protocols, while the second PCR targeted the replication-associated protein (rp) region, designed from a long contig assembled from an influent wastewater sample collected in Gothenburg in 2020.

For both regions, the first-round PCR was performed in a 50 µL reaction consisting of 5 µL cDNA, 1× Taq buffer (Applied Biosystems), 2.25 mM MgCl_2_, 0.2 mM dNTPs (Sigma-Aldrich), 1 U Taq DNA polymerase (Roche, California, USA), 0.3 µM of each primer, and nuclease-free water. Thermal cycling involved initial denaturation at 94 °C for 3 min, followed by 40 cycles of 94 °C for 20 s, annealing at 55 °C (cp region) or 58 °C (rp region) for 30 s, and 72 °C for 1 min, with a final extension at 72 °C for 5 min. The second-round PCR followed the same conditions, but with nested primers. Primer sequences are provided in Table 1.

**Table 1:** Primers and probes used for the detection and amplification of PMMoV

Target	Primer/probe	Primer and probe sequence	Size (bp)	References
qPCR (RP region)	PMMV-FP1	GAGTGGTTTGACCTTAACGTTTGA	68	(Haramoto et al., 2013)
	PMMV-RP1	TTGTCGGTTGCAATGCAAGT		(Zhang et al., 2006)
	PMMV-Probe1	FAM-CCTACCGAAGCAAATG-MGB-NFQ		(Zhang et al., 2006)
PCR1 (CP region)	PMMoV-CP-F1	ATGGCTTACACAGTTTCCAGT	474	(Peng et al., 2015)
	PMMoV-CP-R1	TTAAGGAGTTGTAGCCCAGGTG		(Chung et al., 2012)
	PMMoV-CP-F2	CGTTAGGYAATCAGTTTCAA	313	(Kitajima et al., 2018)
	PMMoV-CP-R2	CGAACTAACTCATTCATGA		(Kitajima et al., 2018)
PCR2 (RP region)	PMMoV-RP-F1	AACGTTTGAGAGGCCTACCG	427	This study
	PMMoV-RP-R1	GCACACGTCGTAGACTCCAA		This study
	PMMoV-RP-F2	GAGAGGCCTACCGAAGCAAA	343	This study
	PMMoV-RP-R2	TTGATACCGCAGCAGAGAGC		This study

For wastewater samples, which typically contained high PMMoV concentrations (Ct < 30), the first-round PCR often yielded clear bands. For lake and raw water samples, where PMMoV concentrations were generally lower (Ct > 30), the nested PCR was necessary to obtain visible amplification. PCR products were examined on 1.5% agarose gels, and bands of the expected size were purified using the QIAquick PCR Purification Kit (Qiagen). Purified amplicons were sequenced by Sanger sequencing at Eurofins Genomics (Ebersberg, Germany).

## Phylogenetic Analysis

PMMoV sequences obtained in this study were manually checked and assembled using BioEdit software (version 7.2.5) (Hall, 1999). Fifty-four available complete PMMoV sequences were downloaded from the NCBI Virus database to serve as reference sequences. For wastewater samples collected between mid-2016 and early-2018, sequences from both the CP (444 bp) and RP (402 bp) regions were aligned with reference genomes using the ClustalW multiple alignment tool implemented in BioEdit. For comparative analysis of PMMoV sequences from wastewater, lake water, and drinking water sources, an additional 320 CP region sequences were downloaded from the NCBI database, resulting in a total of 456 sequences analyzed. Information on the geographic origin of each reference sequence was obtained either from the NCBI record or the origin publication. Phylogenetic trees were inferred using the maximum likelihood method implemented in PhyML 3.0 online platform (http://www.atgc-montpellier.fr/phyml/) (Guindon et al., 2010). The best-fit nucleotide substitution model was automatically selected by the program. All trees were annotated with sample source and country of origins, and visualized using iTOL version 7.2.2 (Letunic & Bork, 2024).

### Data Analysis

PMMoV sequences obtained in this study were categorized into three groups, as wastewater 2016–2017, wastewater 2023–2024, and lake water 2023, based on sample type and collection period. Pairwise genetic distances were calculated within each group to assess intra-group diversity, and then compared across groups using the between-groups mean distance function implemented in MEGA11 software (Tamura et al., 2021). To evaluate whether the genetic distance differences between groups were statistically significant, the Mann-Whitney U test was conducted by using R software (version 4.5.1). A p-value < 0.05 was considered statistically significant.

## Results

### Quantification of PMMoV in Wastewater and Lake Water

**Fig. 1:**
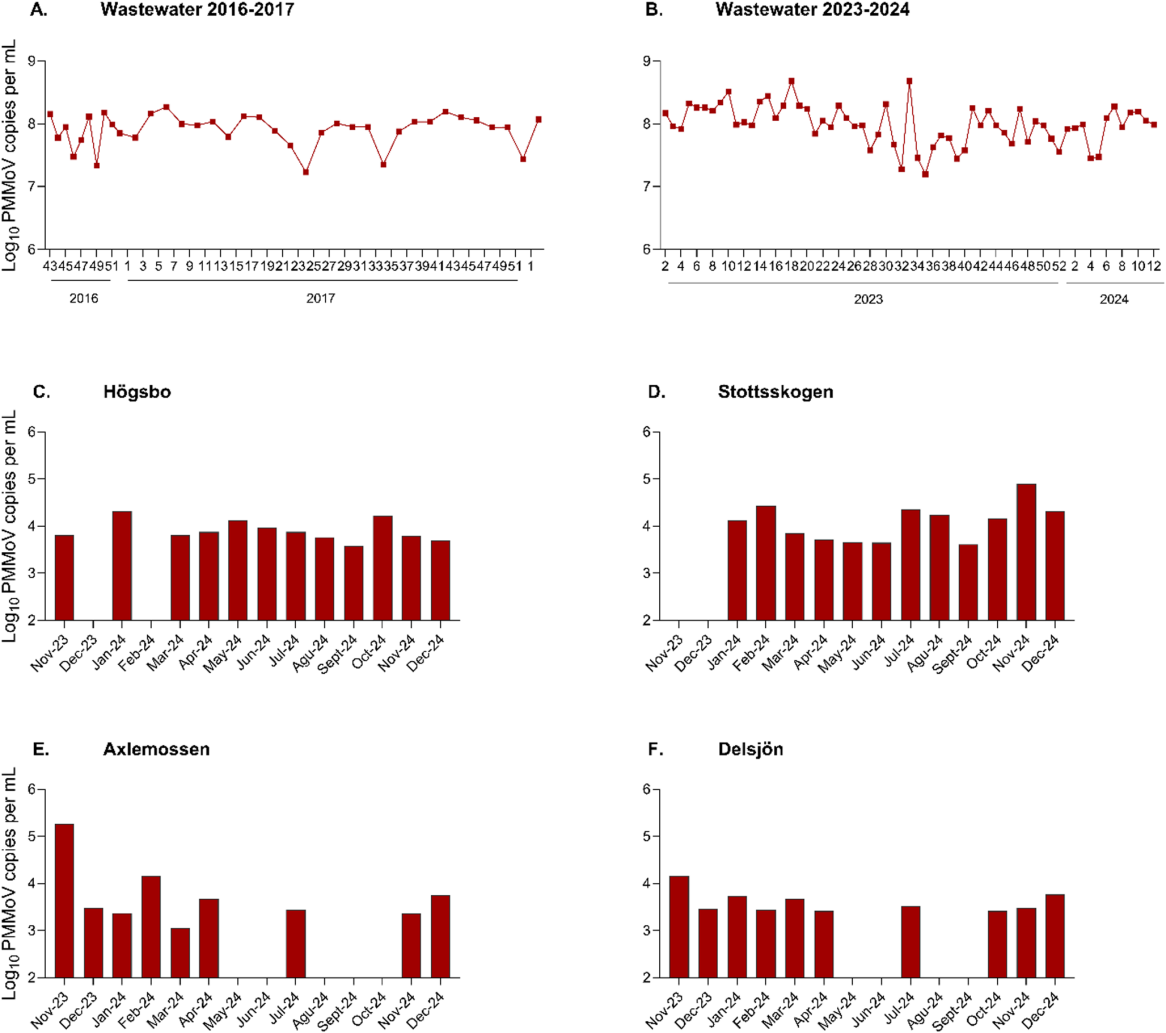
Weekly and monthly PMMoV concentrations (log_10_ genome copies/mL) in Gothenburg, Sweden. **A**–**B** Wastewater influent: **A** mid-2016 to early-2018; **B** 2023–2024. **C**–**F** Lake water (Nov 2023-Dec 2024): **C** Högsbo, **D** Slottsskogen, **E** Axlemossen, **F** Delsjön

PMMoV was consistently detected by qPCR in all wastewater samples collected during both study periods. Concentrations remained relatively stable over time, with mean values of 8.0 and 8.1 log₁₀ genome copies/mL for the mid 2016-early 2018 and 2023–2024 periods, respectively (Table 2; Fig. 1A–B). The overall variation in wastewater concentrations spanned less than 1.5 log₁₀ across the eight-year duration. In lake water, PMMoV concentrations were approximately 3–4 log₁₀ lower than those in wastewater, ranging from 3.1 to 5.3 log₁₀ genome copies/mL (Table 2). The virus was detected in all samples from Högsbo and Slottsskogen, while detection in Axlemossen and Delsjön lakes was less consistent, particularly between May and October (Fig. 1C-F). PMMoV was also detected in 10 out 12 raw water samples used for drinking water production from six DWTPs across Sweden, with concentrations comparable to those in lake water (3.3–5.5 log₁₀ genome copies/mL).

**Table 2:** Summary of PMMoV detection by qPCR and sequencing in Swedish environmental waters

Sample type	Samples tested (*N*)	PMMoV positive (*n*, %)	Concentration means (range; log₁₀ copies/mL)	Sequencing success (*n*, %)
Wastewater (2016-early 2018)	40	37/37 (100%) ^1^	8.0 (7.2–8.3)	36 (90%)
Wastewater (2023–2024)	34	63/63 (100%) ^2^	8.1 (7.2–8.7)	31 (91%)
Lake samples (2023–2024)	55	44/55 (80%)	3.6 (3.1–5.3)	16 (29%)
Source water^3^ (2021–2022)	12	10/12 (83%)	4.5 (3.3–5.5)	2 (17%)

1. Three out of 40 wastewater samples were not quantified due to the lack of materials.
2. Additional 29 weekly wastewater samples from 2023 were added for quantification only but not for sequencing.
3. Drinking water treatment plant inlet.

## Comparison of PMMoV Genotyping Using the CP and RP Regions

To determine which genomic region is more suitable for PMMoV genotyping, two nested PCR systems targeting the coat protein (CP) and replication-associated protein (RP) regions were compared using wastewater samples collected between 2016 and early 2018. The first-round PCR in both systems was sufficiently sensitive to amplify PMMoV from wastewater. PMMoV was successfully amplified in 36 of 40 samples for both regions (Table 2). After Sanger sequencing, 37 sequences were obtained for the CP region (including two replicates of sample WP16-8, used as positive controls) and 35 sequences for the RP region (WP16-1 was excluded due to poor sequence quality).

Among the 37 CP sequences, nucleotide identities ranged from 98.42% to 100%, while the 35 RP sequences showed identities between 97.11% and 100%. The CP sequences displayed only minor nucleotide variations, except for a degenerate base (Y, C/T) at position 198, observed in 46% (17/37) of sequences (Supplementary Fig. 1). This C/T substitution was synonymous and did not alter the amino acid sequence. In contrast, the RP region contained degenerate bases at eight positions across the 402 bp fragment, which could complicate downstream analyses.

**Fig. 2:**
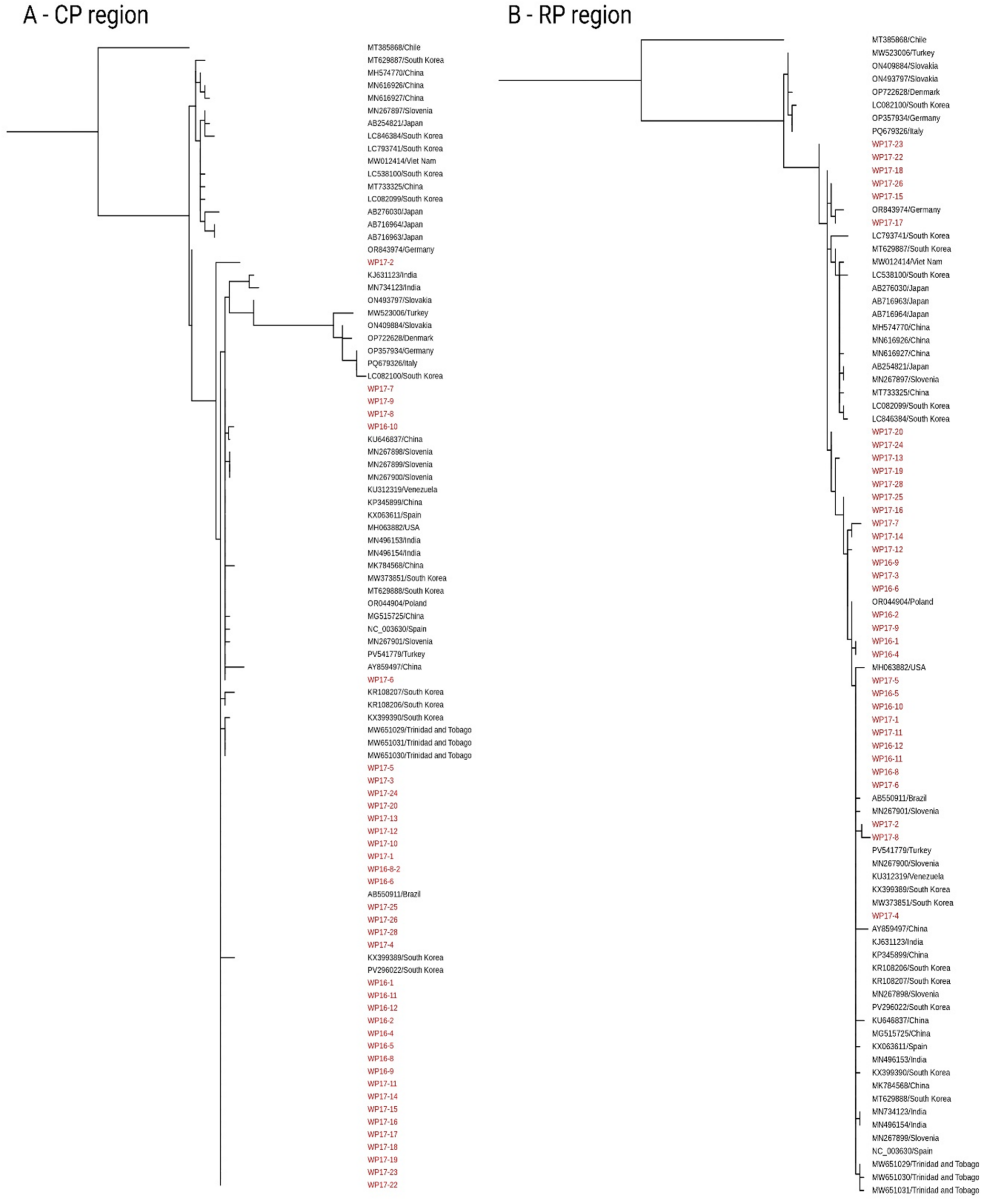
Maximum-likelihood phylogenetic trees of PMMoV sequences from Swedish wastewater (mid-2016 to early-2018). **A** Coat protein (CP) region (444 bp). **B** Replication-associated protein (RP) region (402 bp). Sequences obtained in this study are labeled in red

Phylogenetic trees constructed from both regions revealed no major differences in overall clustering patterns (Fig. 2A and B). In both trees, most local wastewater PMMoV sequences formed a tight clade. A few sequences, such as WP17-2 in the CP tree and WP17-4 in the RP tree, were positioned slightly apart from the main cluster, but the genetic divergence was less than 3%. When compared with global complete reference sequences, PMMoV strains from Asia, Europe, and the Americas were intermixed in both phylogenetic trees. Notably, a large clade consisting mainly of Asian sequences was more distinct in the CP-based tree but merged with the main cluster in the RP-based analysis. Although no major differences were observed between the CP and RP regions, the CP region was selected for further analyses due to fewer degenerate bases in wastewater samples and the availability of more reference sequences.

### Genetic Conservation of PMMoV Strains in Wastewater

To assess potential temporal changes in circulating PMMoV strains in the community, wastewater samples collected during 2023–2024 were analyzed using the CP PCR system. PMMoV was successfully amplified and assembled from 31 of 34 samples. These sequences showed high nucleotide similarity, with identities ranging from 99.55% to 100%, and no amino acid substitutions were detected. The degenerate base (Y, C/T) at position 198 was also observed in 29% (9/31) of the sequences, consistent with earlier findings from 2016 to 2017 wastewater samples. The between-group mean genetic distance between PMMoV sequences from 2016 to 2017 and 2023–2024 was 0.00036, reflecting extremely high nucleotide similarity. A Mann-Whitney U test indicated no statistically significant difference between the two groups (*p* = 0.92), suggesting that the PMMoV population in local wastewater has remained genetically stable over the eight-year period. Phylogenetic analysis further supported this observation, showing no temporal clustering between the two sampling periods (Supplementary Fig. 2).

### Genetic Diversity of PMMoV in Different Environmental Waters and Comparison with Global Strains

To evaluate the genetic variability of PMMoV in different environmental waters, PMMoV was amplified from four lakes located in the same region as the wastewater sampling sites and from raw water used for drinking water production in six DWTPs located in other regions of Sweden. Because viral concentrations in surface and source waters were substantially lower than in wastewater, an additional nested PCR round was needed to amplify the CP region. Despite this, PMMoV sequences could only be successfully assembled from 29% (16/55) of lake samples and 17% (2/12) of source water samples (Table 2). Several samples yielded visible bands in agarose gels, but sequencing quality was poor for reliable assembly.

Among the 16 assembled PMMoV sequences from lake water, nucleotide identities ranged from 96.84% to 100%. The between-group mean genetic distances between PMMoV sequences from lakes and wastewater collected in 2016–2017 and 2023–2024 were 0.00515 and 0.00492, respectively. Mann-Whitney U tests showed no statistically significant difference between lake and wastewater sequences (*p* = 0.43 and *P* = 0.58, respectively), indicating that PMMoV is genetically stable across different water types in the same geographic region. The degenerate base (Y, C/T) previously detected at position 198 in wastewater samples was not observed in any lake or source water sequences. Two sequences from DWTP raw water originated from Ringsjöverket (WP21-098, Lake Bolmen; Supplementary Table 3) and Borgs vattenverk (WP21-078, Motala Stream), showing 97.5% nucleotide similarity.

**Fig. 3:**
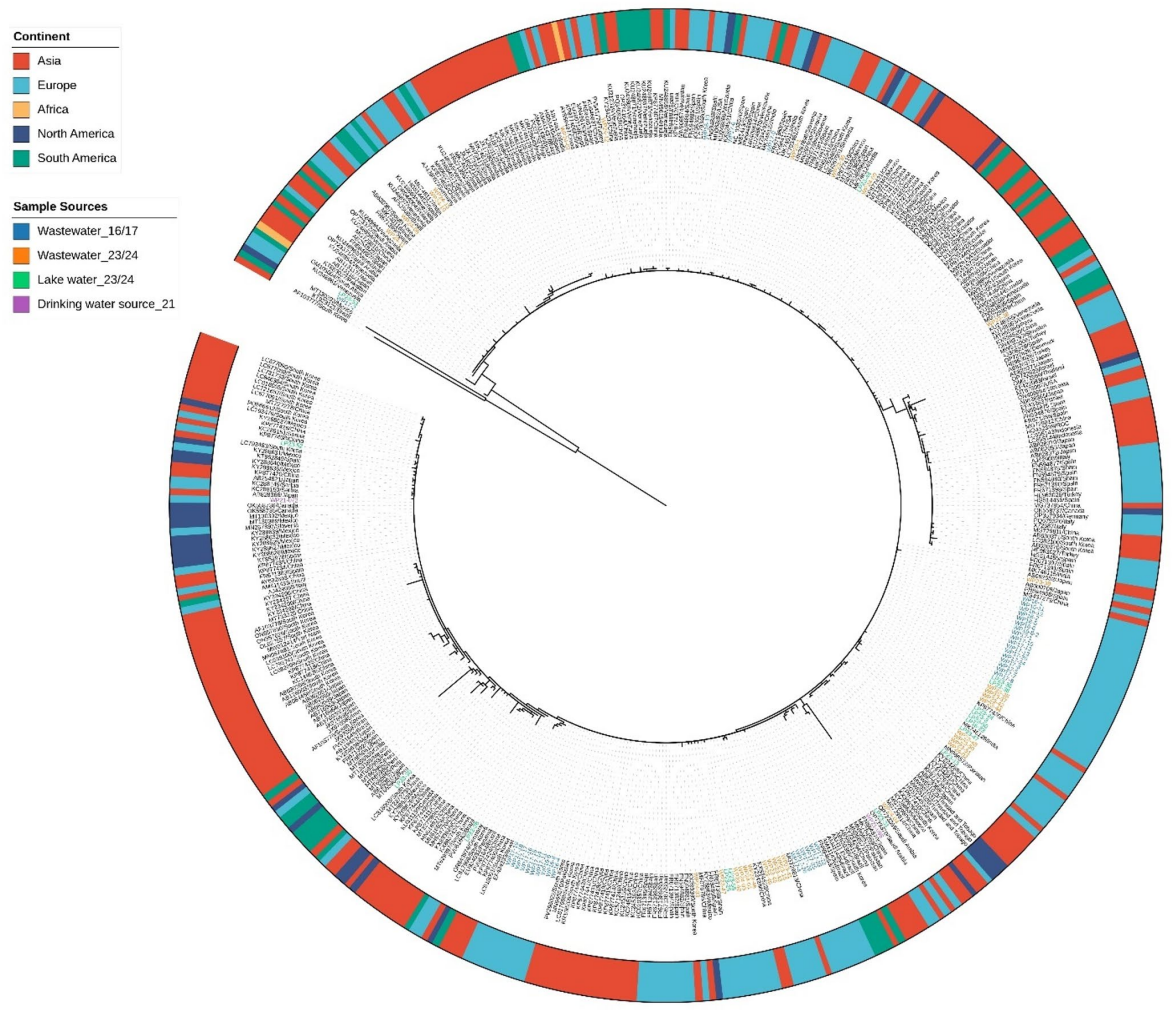
Maximum-likelihood phylogenetic tree of the PMMoV coat protein (CP) region (303 bp). The tree includes all sequences from this study. The sequences are color-coded by sample type, with blue (wastewater, 2016-early 2018), orange (wastewater, 2023–2024), green (lake water, 2023–2024), and purple (drinking water source, 2021). 320 reference sequences from the worldwide were added in the analysis and annotated by the source of origin in the tree

To have a broader view of PMMoV genetic diversity, all CP sequences obtained in this study were aligned with 320 publicly available PMMoV reference sequences from the NCBI database. The phylogenetic analysis revealed that PMMoV sequences from lakes were slightly more divergent than those from wastewater, although most clustered closely together (Fig. 3 and Supplementary Table S4). The two strains from DWTP source waters were separately clustered from the lake and wastewater sequences from Gothenburg, suggesting regional genetic variation within Sweden. Global PMMoV reference sequences were categorized by continent (Asia, Europe, Africa, North America, and South America) and annotated on the phylogenetic tree. Overall, sequences from different continents were highly intermixed, probably due to the low overall genetic diversity of PMMoV. However, several clades with partial regional clustering were observed, particularly among Asian and European strains.

## Discussion

PMMoV was consistently detected across multiple water types in Sweden, including wastewater, lake water, and raw water used for drinking water production, confirming its widespread presence in aquatic environments. Sequence analysis revealed a remarkably high nucleotide similarity among these PMMoV strains, with identities exceeding 96% across all samples. Moreover, the genetic distance between wastewater sequences collected six years apart was extremely low, and no temporal or spatial clustering was detected through phylogenetic analyses. These findings demonstrate that PMMoV strains in Sweden have remained genetically stable over time and across different environmental waters. This observed genetic conservation, combined with its high detection frequency and greater resistance to environmental degradation and water treatment processes compared with other human enteric and respiratory viruses (Burnet et al., 2023; Shirasaki et al., 2020; Zhang et al., 2024), supports its growing use as a viral process indicator for evaluating viral removal efficacy in wastewater and drinking water treatment studies.

On the other hand, the high prevalence and genetic conservation of PMMoV across different water types, especially in lakes, raise concerns about its reliability as a specific viral indicator of human fecal contamination. Although PMMoV is frequently associated with human feces due to the consumption of pepper-containing foods (Persson et al., 2025), several studies have reported its occurrence in feces of animals such as geese, chickens, and seagulls, albeit at concentrations 3–4 logs lower than in human feces (Gyawali et al., 2019; Hamza et al., 2011; Kitajima et al., 2018). Gothenburg is a coastal city with abundant waterfowl and wildlife near sampled lakes, and these animals likely contribute PMMoV to surface waters, potentially leading to overestimation of human fecal input. In addition, agricultural activities may further complicate source attribution. In rural Sweden, manure spreading in farming during spring and autumn is common practice, and runoff from fertilized fields can potentially transport PMMoV and other viral contaminants into streams, lakes, or even drinking water sources. Similar challenges may be magnified in developing countries, where livestock waste is often unmanaged and the animal feces can introduce substantial PMMoV input into surface and groundwater. Altogether, the persistence of PMMoV in the environment and its potential for multiple fecal origins suggest that PMMoV may function more as a general fecal indicator rather than a marker exclusively linked to human fecal contamination. Future studies comparing PMMoV genetic variation in human, agricultural, and wildlife sources will be essential to clarify source attribution and improve its interpretive value in environmental monitoring.

When compared with other conventional and emerging viral indicators for water quality assessment, PMMoV exhibits distinct advantages in terms of its genetic conservation. Traditional coliphages, including somatic and F-specific coliphages, are widely used as viral surrogates and are recommended by international guidelines, such as those from the World Health Organization (WHO) and the European Union Drinking Water Directive, as they better mimic the persistence of enteric viruses in environment waters (Directive, 2020; Organization, 2022). However, their application may be limited by comprising a diverse group of bacteriophages from several viral families, less consistent correlation with the presence and removal of human enteric viruses during water treatment (Singh et al., 2022), and the need of labor-intensive culture-based detection methods. CrAssphage, a bacteriophage that infects *Bacteroides* species, has been proposed as an alternative indicator for human fecal contamination and water quality monitoring, due to its highly specific presence in human feces and is rarely found in animal feces (Sabar et al., 2022). Nevertheless, recent studies have revealed substantial genomic diversity among crAssphage strains, with strains often showing local clustering within specific countries, cities, and even individuals (Edwards et al., 2019; Shkoporov et al., 2019). This high genetic heterogeneity may complicate global comparisons and long-term monitoring. In contrast, our analysis showed that PMMoV has minimal sequence divergence worldwide and maintains consistent detection frequency across both time and geography, making it more suitable for comparative and longitudinal studies of viral removal and treatment efficacy. Meanwhile, other emerging viral indicators, such as cucumber green mottle mosaic virus, tobacco mosaic virus, and bacteriophages with no host presenting during the processing (Saguti et al., 2025; Tandukar et al., 2020; Yasui et al., 2021), are also being evaluated for their potential as viral process indicators in water quality assessments.

The extremely low nucleotide divergence observed among PMMoV sequences worldwide at the CP region in this study contrasts with a previous report suggesting that PMMoV undergoes rapid evolutionary dynamics and has similar evolutionary rates compare to animal RNA viruses (Guan et al., 2018). In general, plant RNA viruses tend to exhibit lower mutation rates and greater genetic conservation because they experience weaker selective pressure within their highly conserved plant hosts, in contrast to animal RNA viruses that replicate in genetically diverse and immunologically complex hosts (Butkovic & González, 2022). The single degenerate base (Y, C/T) detected at the CP region in a large proportion of PMMoV sequences from wastewater, but absent in lake and drinking water source samples, provides a notable insight for further evaluating the utility of PMMoV as a contamination indicator. This heterogeneity in wastewater likely reflects the diversity of the human diet, which aggregates PMMoV strains from varying global food sources and possibly multiple hosts. In contrast, the sequence homogeneity observed in surface waters suggests that more localized and stable non-human sources, such as agricultural runoff or fecal inputs from local wild animals with restricted dietary exposures. Consequently, this specific nucleotide variation could potentially serve as a discriminator between human wastewater pollution and background environmental levels. However, its application for source tracking requires further large-scale comparative investigations across different geographic regions and host species.

Quantitative analysis revealed consistently high and relatively stable PMMoV concentrations in wastewater during the eight-year period, validating its use as a viral process indicator in water treatment evaluation. These favorable characteristics also support the widespread use of PMMoV as a normalization factor for human respiratory and enteric viruses in wastewater-based monitoring. However, these quantitative comparisons between different sampling periods and sample types in this study must be interpreted with caution, since the application of different viral concentration methods and nucleic acid extraction kits may introduce variability in viral recovery efficiency. Future longitudinal studies should prioritize standardized protocols or utilize internal recovery controls, such as Mengovirus, to normalize data across different processing batches and mitigate methodological bias. In addition, potential variations in PMMoV levels, such as the two outliers observed in 2023–2024 and the spatio-temporal variability reported in other studies (Goitom et al., 2024; Maal-Bared et al., 2023), could affect the interpretation of normalized viral targets. Factors including seasonal dietary patterns, tourism, and methodological differences emphasize the need for robust quantification protocols to ensure PMMoV remains a reliable reference marker (Rosengart et al., 2025). The lower PMMoV concentrations detected in surface and source waters, approximately 3–4 log₁₀ lower than in wastewater, are not surprising since these environments receive viral inputs from limited human and animal activities, in contrast to wastewater, which aggregates fecal material from the entire community. These low concentrations reduced sequencing success, indicating that future studies should adopt more sensitive viral concentration and sequencing methods to improve genome recovery from low-titer samples. Nevertheless, the detection of PMMoV across all water types demonstrates that it can serve not only as a stable process indicator in high-viral-load systems like wastewater but also as a sensitive tracer for monitoring viral dissemination in surface waters.

The genetic analysis in this study focused primarily on the CP region, a relatively short fragment commonly used for quantification and pathotyping (Kumari et al., 2023). While this region provides valuable information on PMMoV diversity, it offers limited insight into potential evolutionary changes or genetic variability across other genomic regions. Expanding analyses to include other regions or full-length genomes would enable a more comprehensive evaluation of the suitability of PMMoV as a robust viral indicator. Although the present findings confirm that PMMoV is both abundant and genetically stable across diverse environmental waters in Sweden, international collaborative efforts are still needed to distinguish potential genetic or ecological differences between strains originating from human and non-human sources. Adopting a multi-marker strategy that combines PMMoV with other human-specific viral indicators or bacteriophages will improve the reliability and accuracy of human fecal source tracking in environmental and water quality monitoring.

In conclusion, this study demonstrates that PMMoV is ubiquitously present and genetically stable across different water environments in Sweden. The high sequence conservation observed over eight years and across geographic regions highlights its value as a reliable viral process indicator for evaluating viral removal efficiency in water treatment systems. The detection of PMMoV in lake water, despite the absence of direct wastewater discharge, suggests that environmental runoff or animal contributions may play a role in its distribution. However, this study did not directly analyze animal fecal matter. To further characterize the host range and its impact on water quality assessment, future research should investigate feces from local wildlife, such as geese, seagulls, or rodents, frequently inhabiting these areas. Because non-human sources may limit the reliability of PMMoV as a standalone marker for human fecal contamination, it is recommended integrating it into multi-marker monitoring frameworks. Combining PMMoV with traditional fecal indicators such as *E. coli* and emerging human-specific microbial source tracking markers can enhance the robustness and accuracy of fecal contamination assessments. Overall, this study underscores the importance of genetic-level comparisons in improving our understanding and application of emerging viral indicators in environmental surveillance.

## Supplementary Information

Below is the link to the electronic supplementary material.

Supplementary Material 1.

Supplementary Material 2. Supplementary Material 2. Figure S1. Degenerate nucleotide variation at position 198 of the PMMoV coat protein region in wastewater samples. (A) Multiple sequence alignment of the coat protein region of PMMoV obtained from wastewater samples collected during 2016–2017. The boxed region highlights a degenerate nucleotide (Y, C/T) at position 198, consistently observed across multiple samples. (B) Representative Sanger sequencing chromatograms from selected samples, shown for both forward and reverse reads. The boxed position corresponds to nucleotide 198 and demonstrates overlapping C and T peaks, confirming the presence of a degenerate base rather than a sequencing artifact.

Supplementary Material 3. Supplementary Figure S2. Phylogenetic comparison of PMMoV isolates from wastewater collected in 2016–2017 and 2023–2024. Maximum-likelihood phylogenetic tree based on the coat protein region of PMMoV sequences obtained from wastewater samples. Sequences are color-coded by sampling period, with blue indicating 2016–2017 and orange indicating 2023–2024. Reference sequences from GenBank are shown in black.

## Data Availability

The data and PMMoV sequences obtained in this study are available from the corresponding author upon request.
