# Supplementary material for: Genetic Evaluation of Pepper Mild Mottle Virus as an Indicator in Water Quality Monitoring and Human Fecal Contamination in Swedish Waters
Source: Food Environ Virol. 2026 Apr 17;18(2):16. doi: 10.1007/s12560-026-09690-6 (PMC13090185; doi:10.1007/s12560-026-09690-6)
Supplement: Supplementary file 1 — Supplementary Material 1. [file 12560_2026_9690_MOESM1_ESM.docx]

**Supplementary Tables**

Table S1. Sampling information on archived influent wastewater.

| **Sample Name** | **Year** | **Week** | **Period** | **Flow (m^3^/s)** |
| --- | --- | --- | --- | --- |
| WP16-1 | 2016 | 40 | 3/10-10/10 | / |
| WP16-2 | 2016 | 41 | 10/10-17/10 | / |
| WP16-3 | 2016 | 42 | 17/10-24/10 | / |
| WP16-4 | 2016 | 43 | 24/10-31/10 | 2 |
| WP16-5 | 2016 | 44 | 31/10-7/11 | 3.6 |
| WP16-6 | 2016 | 45 | 7/11-14/11 | 2.4 |
| WP16-7 | 2016 | 46 | 14/11-21/11 | 4.5 |
| WP16-8 | 2016 | 47 | 21/11-28/11 | 2.9 |
| WP16-9 | 2016 | 48 | 28/11-5/12 | 2.2 |
| WP16-10 | 2016 | 49 | 5/12-12/12 | 3.1 |
| WP16-11 | 2016 | 50 | 12/12-19/12 | 2.3 |
| WP16-12 | 2016 | 51 | 19/12-26/12 | 2.8 |
| WP17-1 | 2016 | 52 | 26/12-2/1 | 2.75 |
| WP17-2 | 2017 | 2 | 9/1-16/1 | 4.2 |
| WP17-3 | 2017 | 4 | 23/1-30/1 | 2.3 |
| WP17-4 | 2017 | 6 | 6/2-13/2 | 1.9 |
| WP17-5 | 2017 | 8 | 20/2-27/2 | 1.9 |
| WP17-6 | 2017 | 10 | 6/3-13/3 | 2.9 |
| WP17-7 | 2017 | 12 | 20/3-27/3 | 2.9 |
| WP17-8 | 2017 | 14 | 3/4-10/4 | 2.2 |
| WP17-9 | 2017 | 16 | 17/4-24/4 | 1.9 |
| WP17-10 | 2017 | 18 | 1/5-8/5 | 2.1 |
| WP17-11 | 2017 | 20 | 15/5-22/5 | 2 |
| WP17-12 | 2017 | 22 | 29/5-5/6 | 2.6 |
| WP17-13 | 2017 | 24 | 12/6-19/6 | 2.8 |
| WP17-14 | 2017 | 26 | 26/6-3/7 | 2 |
| WP17-15 | 2017 | 28 | 10/7-17/7 | 2.6 |
| WP17-16 | 2017 | 30 | 24/7-31/7 | 4.2 |
| WP17-17 | 2017 | 31 | 7/8-14/8 | 2 |
| WP17-18 | 2017 | 32 | 21/8-28/8 | 2 |
| WP17-19 | 2017 | 34 | 4/9-11/9 | 4 |
| WP17-20 | 2017 | 36 | 18/9-25/9 | 3 |
| WP17-21 | 2017 | 38 | 2/10-9/10 | 3.5 |
| WP17-22 | 2017 | 40 | 16/10-23/10 | 3 |
| WP17-23 | 2017 | 42 | 30/10-6/11 | 3 |
| WP17-24 | 2017 | 44 | 13/11-20/11 | 3.2 |
| WP17-25 | 2017 | 46 | 27/11-4/12 | 4.2 |
| WP17-26 | 2017 | 48 | 11/12-18/12 | 4 |
| WP17-27 | 2017 | 50 | 25/12-1/1 2018 | 6 |
| WP17-28 | 2018 | 2 | 1/8-1/15 | 4.24 |
| WP23-31 | 2023 | 32 | 07/08-14/08 | 3.42 |
| WP23-32 | 2023 | 33 | 14/08-21/08 | 3.66 |
| WP23-33 | 2023 | 34 | 21/08-28/08 | 4.87 |
| WP23-34 | 2023 | 35 | 28/08-04/09 | 4.29 |
| WP23-35 | 2023 | 36 | 04/09-11/09 | 2.93 |
| WP23-36 | 2023 | 37 | 11/09-18/09 | 2.91 |
| WP23-37 | 2023 | 38 | 18/09-23/09 | 5.26 |
| WP23-38 | 2023 | 39 | 25/09-02/10 | 3.69 |
| WP23-39 | 2023 | 40 | 02/10-09/10 | 5.92 |
| WP23-40 | 2023 | 41 | 09/10-16/10 | 5.32 |
| WP23-41 | 2023 | 42 | 16/10-23/10 | 4.41 |
| WP23-42 | 2023 | 43 | 23/10-30/10 | 4.5 |
| WP23-43 | 2023 | 44 | 30/10-06/11 | 5.74 |
| WP23-44 | 2023 | 45 | 06/11-13/11 | 5.04 |
| WP23-45 | 2023 | 46 | 13/11-20/11 | 3.36 |
| WP23-46 | 2023 | 47 | 20/11-27/11 | 3.54 |
| WP23-47 | 2023 | 48 | 27/11-04/12 | 2.97 |
| WP23-48 | 2023 | 49 | 04/12-11/12 | 2.95 |
| WP23-49 | 2023 | 50 | 11/12-18/12 | 3.55 |
| WP23-50 | 2023 | 51 | 18/12-25/12 | 6.81 |
| WP23-51 | 2023 | 52 | 25/12-01/01 | 7.1 |
| WP24-1 | 2024 | 1 | 01/01-08/01 | 4.71 |
| WP24-2 | 2024 | 2 | 08/01-15/01 | 3.18 |
| WP24-3 | 2024 | 3 | 15/01-22/01 | 3.63 |
| WP24-4 | 2024 | 4 | 22/01-29/01 | 9.5 |
| WP24-5 | 2024 | 5 | 29/01-05/02 | 6.5 |
| WP24-6 | 2024 | 6 | 05/02-12/02 | / |
| WP24-7 | 2024 | 7 | 12/02-19/02 | 7.48 |
| WP24-8 | 2024 | 8 | 19/02-26/02 | 8.2 |
| WP24-9 | 2024 | 9 | 26/02-04/03 | 5.69 |
| WP24-10 | 2024 | 10 | 04/03-11/03 | 3.66 |
| WP24-11 | 2024 | 11 | 11/03-18/03 | 4.67 |
| WP24-12 | 2024 | 12 | 18/03-25/03 | 4.31 |
| WP24-13 | 2024 | 13 | 25/03-01/04 | 4.62 |

Table S2. Sampling information on lake samples.

| **Sample ID** | **Collection sites** | **Week of collection** | **Date** | **Total volume (mL)** |
| --- | --- | --- | --- | --- |
| LP23-1 | Axelmossen | 46 | 11/15/2023 | 1000 |
| LP23-2 | Delsjön-1 | 46 | 11/16/2023 | 900 |
| LP23-3 | Delsjön-2 | 46 | 11/16/2023 | 950 |
| LP23-4 | Högsbo (Torpadammen) | 46 | 11/16/2023 | 950 |
| LP23-5 | Delsjön | 50 | 12/16/2023 | 1000 |
| LP23-6 | Axelmossen | 50 | 12/18/2023 | 1000 |
| LP23-7 | Högsbo (Torpadammen) | 50 | 12/18/2023 | 950 |
| LP23-8 | Slottsskogen | 3 | 1/18/2024 | 950 |
| LP23-9 | Axelmossen | 3 | 1/18/2024 | 1000 |
| LP23-10 | Delsjön | 4 | 1/25/2024 | 1000 |
| LP23-11 | Högsbo (Torpadammen) | 4 | 1/25/2024 | 1000 |
| LP23-12 | Slottsskogen | 7 | 2/13/2024 | 1000 |
| LP23-13 | Högsbo (Torpadammen) | 7 | 2/15/2024 | 950 |
| LP23-14 | Delsjön | 7 | 2/17/2024 | 1000 |
| LP23-15 | Axelmossen | 7 | 2/20/2024 | 1000 |
| LP23-16 | Slottsskogen | 11 | 3/11/2024 | 1000 |
| LP23-17 | Högsbo (Torpadammen) | 11 | 3/11/2024 | 950 |
| LP23-18 | Delsjön | 11 | 3/13/2024 | 1000 |
| LP23-19 | Axelmossen | 11 | 3/13/2024 | 1000 |
| LP23-20 | Högsbo (Torpadammen) | 15 | 4/11/2024 | 1000 |
| LP23-21 | Delsjön | 15 | 4/14/2024 | 1000 |
| LP23-22 | Slottsskogen | 16 | 4/15/2024 | 1000 |
| LP23-23 | Axelmossen | 16 | 4/17/2024 | 1000 |
| LP23-24 | Delsjön | 20 | 5/14/2024 | 1000 |
| LP23-25 | Slottsskogen | 20 | 5/15/2024 | 2000 |
| LP23-26 | Axlemossen | 21 | 5/20/2024 | 1000 |
| LP23-27 | Högsbo (Torpadammen) | 21 | 5/21/2024 | 1000 |
| LP23-28 | Högsbo (Torpadammen) | 25 | 2024/6/17 | 1000 |
| LP23-29 | Delsjön | 25 | 2024/6/17 | 1000 |
| LP23-30 | Axlemossen | 25 | 2024/6/19 | 1000 |
| LP23-31 | Slottsskogen | 25 | 2024/6/19 | 1000 |
| LP23-32 | Axlemossen | 29 | 2024/7/19 | 1000 |
| LP23-33 | Slottsskogen | 30 | 2024/7/23 | 1000 |
| LP23-34 | Delsjön | 30 | 2024/7/24 | 1000 |
| LP23-35 | Högsbo (Torpadammen) | 30 | 2024/7/25 | 1000 |
| LP23-36 | Slottsskogen | 34 | 2024/8/21 | 900 |
| LP23-37 | Högsbo (Torpadammen) | 35 | 2024/8/28 | 1000 |
| LP23-38 | Axlemossen | 35 | 2024/8/28 | 1000 |
| LP23-39 | Delsjön | 35 | 2024/8/29 | 1000 |
| LP23-40 | Högsbo (Torpadammen) | 38 | 2024/9/19 | 1000 |
| LP23-41 | Axlemossen | 38 | 2024/9/19 | 1000 |
| LP23-42 | Delsjön | 38 | 2024/9/19 | 1000 |
| LP23-43 | Slottsskogen | 38 | 2024/9/19 | 1000 |
| LP23-44 | Delsjön | 42 | 2024/10/16 | 1000 |
| LP23-45 | Axlemossen | 42 | 2024/10/17 | 1000 |
| LP23-46 | Högsbo (Torpadammen) | 43 | 2024/10/23 | 1000 |
| LP23-47 | Slottsskogen | 43 | 2024/10/23 | 1000 |
| LP23-48 | Högsbo (Torpadammen) | 47 | 2024/11/19 | 1000 |
| LP23-49 | Axlemossen | 47 | 2024/11/19 | 1000 |
| LP23-50 | Slottsskogen | 47 | 2024/11/20 | 1000 |
| LP23-51 | Delsjön | 47 | 2024/11/20 | 1000 |
| LP23-52 | Högsbo (Torpadammen) | 51 | 2024/12/17 | 1000 |
| LP23-53 | Axlemossen | 51 | 2024/12/17 | 1000 |
| LP23-54 | Delsjön | 51 | 2024/12/20 | 1000 |
| LP23-55 | Slottsskogen | 51 | 2024/12/20 | 1000 |

Table S3. Sampling information on raw water used for drinking water production in Sweden.

| **Sample ID** | **Drinking water**  **treatment plant** | **Source water** | **Collection time** | **Volume (L)** |
| --- | --- | --- | --- | --- |
| Raw-1 | Lovö vattenverk | Lake Mälaren | March-2021 | 20 |
| Raw-2 |  |  | December-2021 | 20 |
| Raw-3 | Görvälnverket | Lake Mälaren | March-2021 | 20 |
| Raw-4 |  |  | October-2021 | 20 |
| Raw-5 | Borgs vattenverk | Motala Stream | April-2021 | 370 |
| Raw-6 |  |  | February-2022 | 1326 |
| Raw-7 | Kvarnagårdens vattenverk | Lake Neden (80%) and  ground water (20%) | March-2021 | 150 |
| Raw-8 |  |  | Januray-2022 | 153 |
| Raw-9 | Vombverket | Lake Vomb | April-2021 | 255 |
| Raw-10 |  |  | Feburary-2022 | 454 |
| Raw-11 | Ringsjöverket | Lake Bolmen | March-2021 | 7085 |
| Raw-12 |  |  | November-2021 | 181 |

Table S4. Pairwise nucleotide p-distances between Swedish PMMoV sequences obtained in this study and 8 selected global reference sequences at CP region.

|  | NC_003630  Spain | LC846384  South Korea | MW651029  Trinidad and Tobago | MW523006  Turkey | MT385868  Chile | MN267897 Slovenia | MH574770  China | KU312319 Venezuela |
| --- | --- | --- | --- | --- | --- | --- | --- | --- |
| WP16-1 | 0.003 | 0.023 | 0.003 | 0.043 | 0.066 | 0.017 | 0.017 | 0.000 |
| WP16-10 | 0.003 | 0.026 | 0.007 | 0.046 | 0.066 | 0.020 | 0.017 | 0.000 |
| WP16-11 | 0.003 | 0.023 | 0.003 | 0.043 | 0.066 | 0.017 | 0.017 | 0.000 |
| WP16-12 | 0.003 | 0.023 | 0.003 | 0.043 | 0.066 | 0.017 | 0.017 | 0.000 |
| WP16-2 | 0.003 | 0.023 | 0.003 | 0.043 | 0.066 | 0.017 | 0.017 | 0.000 |
| WP16-4 | 0.007 | 0.023 | 0.003 | 0.043 | 0.069 | 0.017 | 0.020 | 0.003 |
| WP16-5 | 0.003 | 0.023 | 0.003 | 0.043 | 0.066 | 0.017 | 0.017 | 0.000 |
| WP16-6 | 0.003 | 0.023 | 0.003 | 0.043 | 0.066 | 0.017 | 0.017 | 0.000 |
| WP16-8 | 0.007 | 0.023 | 0.003 | 0.043 | 0.069 | 0.017 | 0.020 | 0.003 |
| WP16-8-2 | 0.003 | 0.023 | 0.003 | 0.043 | 0.066 | 0.017 | 0.017 | 0.000 |
| WP16-9 | 0.007 | 0.023 | 0.003 | 0.043 | 0.069 | 0.017 | 0.020 | 0.003 |
| WP17-1 | 0.003 | 0.023 | 0.003 | 0.043 | 0.066 | 0.017 | 0.017 | 0.000 |
| WP17-10 | 0.003 | 0.023 | 0.003 | 0.043 | 0.066 | 0.017 | 0.017 | 0.000 |
| WP17-11 | 0.007 | 0.023 | 0.003 | 0.043 | 0.069 | 0.017 | 0.020 | 0.003 |
| WP17-12 | 0.003 | 0.023 | 0.003 | 0.043 | 0.066 | 0.017 | 0.017 | 0.000 |
| WP17-13 | 0.003 | 0.023 | 0.003 | 0.043 | 0.066 | 0.017 | 0.017 | 0.000 |
| WP17-14 | 0.007 | 0.023 | 0.003 | 0.043 | 0.069 | 0.017 | 0.020 | 0.003 |
| WP17-15 | 0.007 | 0.023 | 0.003 | 0.043 | 0.069 | 0.017 | 0.020 | 0.003 |
| WP17-16 | 0.007 | 0.023 | 0.003 | 0.043 | 0.069 | 0.017 | 0.020 | 0.003 |
| WP17-17 | 0.007 | 0.023 | 0.003 | 0.043 | 0.069 | 0.017 | 0.020 | 0.003 |
| WP17-18 | 0.007 | 0.023 | 0.003 | 0.043 | 0.069 | 0.017 | 0.020 | 0.003 |
| WP17-19 | 0.007 | 0.023 | 0.003 | 0.043 | 0.069 | 0.017 | 0.020 | 0.003 |
| WP17-2 | 0.022 | 0.037 | 0.022 | 0.055 | 0.077 | 0.030 | 0.030 | 0.018 |
| WP17-20 | 0.003 | 0.023 | 0.003 | 0.043 | 0.066 | 0.017 | 0.017 | 0.000 |
| WP17-22 | 0.007 | 0.023 | 0.003 | 0.043 | 0.069 | 0.017 | 0.020 | 0.003 |
| WP17-23 | 0.007 | 0.023 | 0.003 | 0.043 | 0.069 | 0.017 | 0.020 | 0.003 |
| WP17-24 | 0.003 | 0.023 | 0.003 | 0.043 | 0.066 | 0.017 | 0.017 | 0.000 |
| WP17-25 | 0.007 | 0.023 | 0.003 | 0.043 | 0.069 | 0.017 | 0.020 | 0.003 |
| WP17-26 | 0.007 | 0.023 | 0.003 | 0.043 | 0.069 | 0.017 | 0.020 | 0.003 |
| WP17-28 | 0.007 | 0.023 | 0.003 | 0.043 | 0.069 | 0.017 | 0.020 | 0.003 |
| WP17-3 | 0.003 | 0.023 | 0.003 | 0.043 | 0.066 | 0.017 | 0.017 | 0.000 |
| WP17-4 | 0.007 | 0.023 | 0.003 | 0.043 | 0.069 | 0.017 | 0.020 | 0.003 |
| WP17-5 | 0.003 | 0.023 | 0.003 | 0.043 | 0.066 | 0.017 | 0.017 | 0.000 |
| WP17-6 | 0.003 | 0.026 | 0.007 | 0.046 | 0.066 | 0.020 | 0.017 | 0.000 |
| WP17-7 | 0.003 | 0.023 | 0.003 | 0.043 | 0.066 | 0.017 | 0.017 | 0.000 |
| WP17-8 | 0.003 | 0.023 | 0.003 | 0.043 | 0.066 | 0.017 | 0.017 | 0.000 |
| WP17-9 | 0.003 | 0.026 | 0.007 | 0.046 | 0.066 | 0.020 | 0.017 | 0.000 |
| WP23-1 | 0.003 | 0.026 | 0.007 | 0.046 | 0.066 | 0.020 | 0.017 | 0.000 |
| WP23-15 | 0.007 | 0.023 | 0.003 | 0.043 | 0.069 | 0.017 | 0.020 | 0.003 |
| WP23-35 | 0.003 | 0.023 | 0.003 | 0.043 | 0.066 | 0.017 | 0.017 | 0.000 |
| WP23-36 | 0.003 | 0.026 | 0.007 | 0.046 | 0.066 | 0.020 | 0.017 | 0.000 |
| WP23-37 | 0.003 | 0.023 | 0.003 | 0.043 | 0.066 | 0.017 | 0.017 | 0.000 |
| WP23-38 | 0.003 | 0.026 | 0.007 | 0.046 | 0.066 | 0.020 | 0.017 | 0.000 |
| WP23-39 | 0.003 | 0.026 | 0.007 | 0.046 | 0.066 | 0.020 | 0.017 | 0.000 |
| WP23-40 | 0.007 | 0.023 | 0.003 | 0.043 | 0.070 | 0.017 | 0.020 | 0.003 |
| WP23-41 | 0.007 | 0.023 | 0.003 | 0.043 | 0.069 | 0.017 | 0.020 | 0.003 |
| WP23-42 | 0.003 | 0.023 | 0.003 | 0.043 | 0.066 | 0.017 | 0.017 | 0.000 |
| WP23-43 | 0.007 | 0.023 | 0.003 | 0.043 | 0.069 | 0.017 | 0.020 | 0.003 |
| WP23-44 | 0.003 | 0.023 | 0.003 | 0.043 | 0.066 | 0.017 | 0.017 | 0.000 |
| WP23-45 | 0.007 | 0.023 | 0.003 | 0.043 | 0.069 | 0.017 | 0.020 | 0.003 |
| WP23-46 | 0.007 | 0.023 | 0.003 | 0.043 | 0.069 | 0.017 | 0.020 | 0.003 |
| WP23-47 | 0.007 | 0.023 | 0.003 | 0.043 | 0.069 | 0.017 | 0.020 | 0.003 |
| WP23-48 | 0.003 | 0.023 | 0.003 | 0.043 | 0.066 | 0.017 | 0.017 | 0.000 |
| WP23-49 | 0.007 | 0.023 | 0.003 | 0.043 | 0.069 | 0.017 | 0.020 | 0.003 |
| WP23-50 | 0.003 | 0.023 | 0.003 | 0.043 | 0.066 | 0.017 | 0.017 | 0.000 |
| WP23-51 | 0.003 | 0.023 | 0.003 | 0.043 | 0.066 | 0.017 | 0.017 | 0.000 |
| WP24-01 | 0.007 | 0.023 | 0.003 | 0.043 | 0.069 | 0.017 | 0.020 | 0.003 |
| WP24-02 | 0.003 | 0.023 | 0.003 | 0.043 | 0.066 | 0.017 | 0.017 | 0.000 |
| WP24-03 | 0.007 | 0.023 | 0.003 | 0.043 | 0.069 | 0.017 | 0.020 | 0.003 |
| WP24-04 | 0.003 | 0.023 | 0.003 | 0.043 | 0.066 | 0.017 | 0.017 | 0.000 |
| WP24-05 | 0.007 | 0.023 | 0.003 | 0.043 | 0.069 | 0.017 | 0.020 | 0.003 |
| WP24-06 | 0.003 | 0.026 | 0.007 | 0.046 | 0.066 | 0.020 | 0.017 | 0.000 |
| WP24-07 | 0.003 | 0.026 | 0.007 | 0.046 | 0.066 | 0.020 | 0.017 | 0.000 |
| WP24-09 | 0.003 | 0.026 | 0.007 | 0.046 | 0.066 | 0.020 | 0.017 | 0.000 |
| WP24-10 | 0.003 | 0.026 | 0.007 | 0.046 | 0.066 | 0.020 | 0.017 | 0.000 |
| WP24-11 | 0.003 | 0.026 | 0.007 | 0.046 | 0.066 | 0.020 | 0.017 | 0.000 |
| WP24-12 | 0.003 | 0.026 | 0.007 | 0.046 | 0.066 | 0.020 | 0.017 | 0.000 |
| WP24-13 | 0.003 | 0.026 | 0.007 | 0.046 | 0.066 | 0.020 | 0.017 | 0.000 |
| LP23-8 | 0.003 | 0.028 | 0.007 | 0.045 | 0.069 | 0.021 | 0.017 | 0.000 |
| LP23-15 | 0.004 | 0.030 | 0.004 | 0.035 | 0.074 | 0.022 | 0.026 | 0.004 |
| LP16-23 | 0.008 | 0.035 | 0.012 | 0.046 | 0.081 | 0.027 | 0.023 | 0.004 |
| LP23-24 | 0.004 | 0.028 | 0.007 | 0.046 | 0.070 | 0.021 | 0.018 | 0.000 |
| LP23-25 | 0.010 | 0.026 | 0.007 | 0.046 | 0.073 | 0.020 | 0.023 | 0.007 |
| LP23-28 | 0.026 | 0.030 | 0.023 | 0.056 | 0.076 | 0.023 | 0.026 | 0.023 |
| LP23-30 | 0.017 | 0.021 | 0.007 | 0.045 | 0.076 | 0.014 | 0.017 | 0.014 |
| LP23-31 | 0.008 | 0.036 | 0.012 | 0.047 | 0.083 | 0.028 | 0.024 | 0.004 |
| LP23-36 | 0.003 | 0.027 | 0.007 | 0.044 | 0.068 | 0.020 | 0.017 | 0.000 |
| LP23-38 | 0.003 | 0.023 | 0.007 | 0.046 | 0.066 | 0.017 | 0.017 | 0.000 |
| LP23-44 | 0.010 | 0.033 | 0.013 | 0.053 | 0.073 | 0.026 | 0.023 | 0.007 |
| LP23-47 | 0.010 | 0.028 | 0.014 | 0.045 | 0.070 | 0.021 | 0.017 | 0.007 |
| LP23-50 | 0.003 | 0.028 | 0.007 | 0.045 | 0.069 | 0.021 | 0.017 | 0.000 |
| LP23-52 | 0.024 | 0.007 | 0.021 | 0.051 | 0.075 | 0.000 | 0.010 | 0.021 |
| LP23-53 | 0.008 | 0.028 | 0.004 | 0.036 | 0.075 | 0.020 | 0.024 | 0.004 |
| LP23-55 | 0.007 | 0.024 | 0.003 | 0.042 | 0.073 | 0.017 | 0.021 | 0.003 |
| WP21-072  raw water | 0.023 | 0.007 | 0.020 | 0.053 | 0.073 | 0.000 | 0.010 | 0.020 |
| WP21-098  raw water | 0.014 | 0.031 | 0.010 | 0.048 | 0.079 | 0.024 | 0.027 | 0.010 |
