# Supplementary figures and images for: Genetic Evaluation of Pepper Mild Mottle Virus as an Indicator in Water Quality Monitoring and Human Fecal Contamination in Swedish Waters
Source: Food Environ Virol. 2026 Apr 17;18(2):16. doi: 10.1007/s12560-026-09690-6 (PMC13090185; doi:10.1007/s12560-026-09690-6)

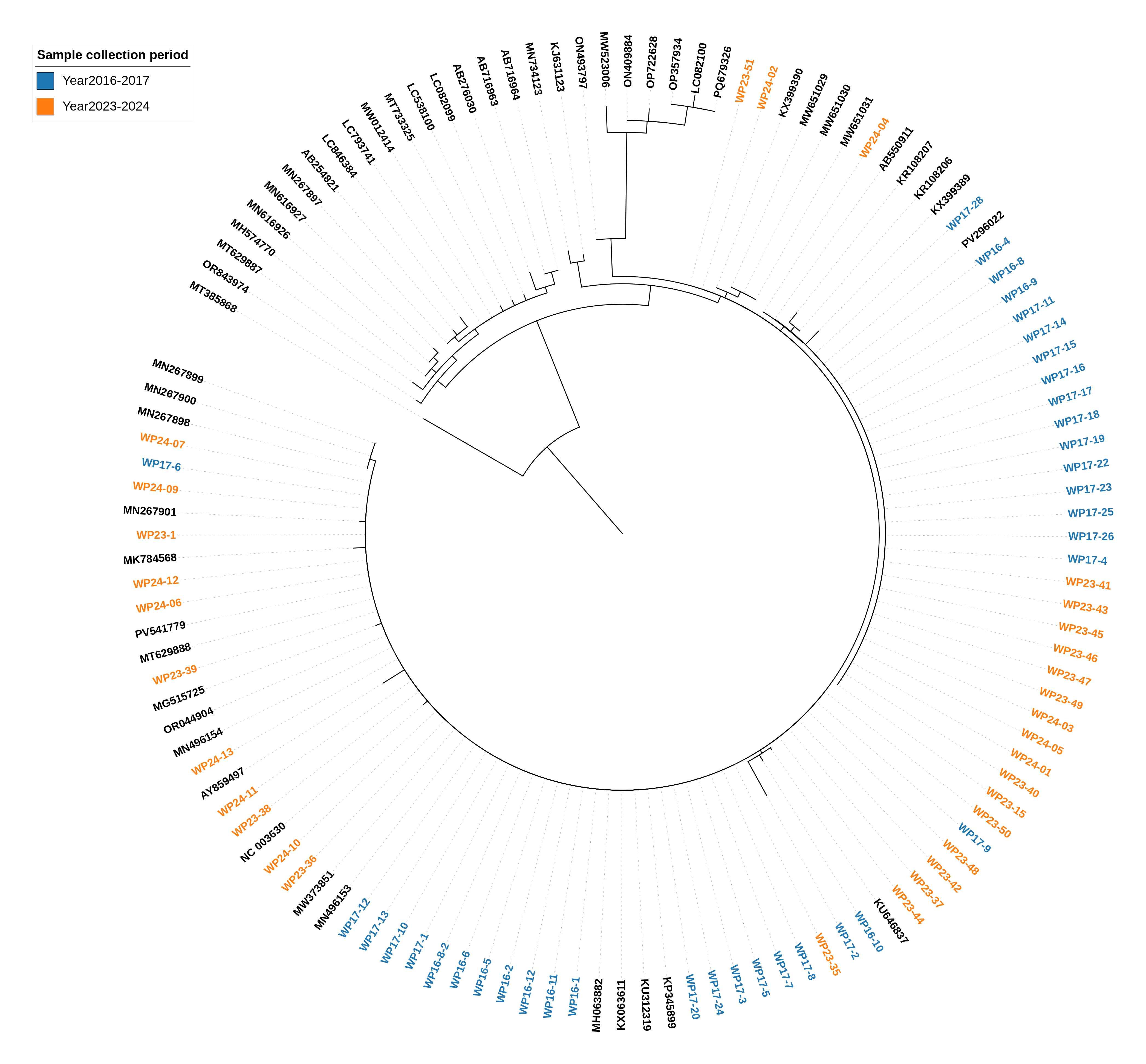

Supplement: Supplementary file 2 — Supplementary Material 2. Supplementary Material 2. Figure S1. Degenerate nucleotide variation at position 198 of the PMMoV coat protein region in wastewater samples. (A) Multiple sequence alignment of the coat protein region of PMMoV obtained from wastewater samples collected during 2016–2017. The boxed region highlights a degenerate nucleotide (Y, C/T) at position 198, consistently observed across multiple samples. (B) Representative Sanger sequencing chromatograms from selected samples, shown for both forward and reverse reads. The boxed position corresponds to nucleotide 198 and demonstrates overlapping C and T peaks, confirming the presence of a degenerate base rather than a sequencing artifact. [file 12560_2026_9690_MOESM2_ESM.jpg]

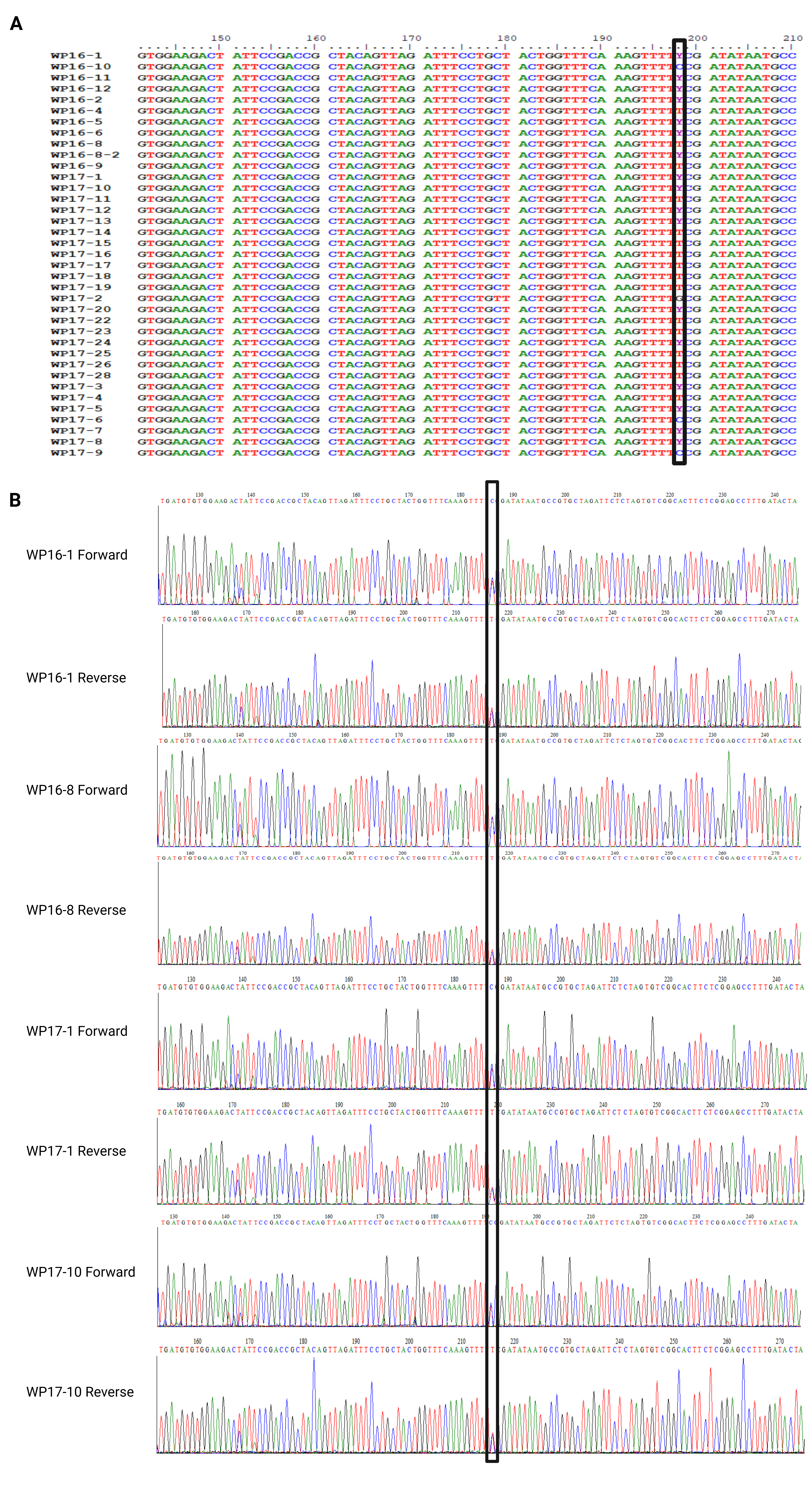

Supplement: Supplementary file 3 — Supplementary Material 3. Supplementary Figure S2. Phylogenetic comparison of PMMoV isolates from wastewater collected in 2016–2017 and 2023–2024. Maximum-likelihood phylogenetic tree based on the coat protein region of PMMoV sequences obtained from wastewater samples. Sequences are color-coded by sampling period, with blue indicating 2016–2017 and orange indicating 2023–2024. Reference sequences from GenBank are shown in black. [file 12560_2026_9690_MOESM3_ESM.jpeg]
